# Effects of Photobiomodulation in Children with Down Syndrome and Possible Sleep Bruxism: Protocol For A Randomized, Controlled, Blind, Clinical Trial: Study protocol clinical trial (SPIRIT compliant)

**DOI:** 10.1097/MD.0000000000019904

**Published:** 2020-04-24

**Authors:** Mônica da Consolação Canuto Salgueiro, Tamiris Silva, Lara Jansiski Motta, Anna Carolina Ratto Tempestini Horliana, Marcela Letícia Leal Gonçalves, Andréa Oliver Gomes, Marcelo Mendes Pinto, Carolina Carvalho Bortoletto, Olga Maria Altavista, Alessandro Melo Deana, Daniela de Fátima Teixeira da Silva, Elaine Marcilio Santos, Paula Midori Castelo, Kristianne Porta Santos Fernandes, Raquel Agnelli Mesquita-Ferrari, Sandra Kalil Bussadori

**Affiliations:** a Universidade Nove de Julho, UNINOVE, São Paulo; b Universidade Metropolitana de Santos, UNIMES, Santos; c Universidade Federal de São Paulo (UNIFESP), Diadema, SP, Brazil.

**Keywords:** acupoints., Bruxism, children, down syndrome, low level laser therapy, photobiomodulation

## Abstract

Hypotonia, particularly of the masticatory and oropharyngeal muscles, is 1 of the main characteristics of Down Syndrome (DS), resulting in impaired speech, chewing and swallowing. Moreover, the complete or partial obstruction of the airways during sleep may occur due to hypotonia of the tongue, leading to snoring and sleep disorders, such as obstructive apnea and sleep bruxism.

**Objective:**

Analyze salivary levels of dopamine and cortisol and muscle activity before and after treatment with low-level laser therapy administered to acupoints in children with DS.

**Methods:**

A randomized, controlled, clinical trial will be conducted. Individuals 4 to 17 years of age with a diagnosis of DS and possible sleep bruxism will be screened at the Integrated Health Clinic of Nove de Julho University. We will evaluate orofacial dysfunction (Nordic Orofacial Test - Screening questionnaire), Masseter muscle activity during sleep will be assessed by BiteStrip and the masticatory muscles will be evaluated by electromyography (BTS TMJOINT) head posture as well as salivary cortisol and dopamine. After the evaluations, the participants will be randomized into 2 groups: Grupo 1 – treatment with low-level laser therapy at a wavelength of 808 nm; Group 2 – sham treatment (simulated laser therapy). Treatment will be conducted twice per week for a total of 12 sessions. The data will be tabulated and treated using GraphPad Prism version 7.0. The Kolmogorov-Smirnov test will be used to determine the normality of the data. Variables that fit the Gaussian curve will be expressed as mean and standard deviation. The ANOVA 2-way will be used for comparisons between the groups, with the significance level set to 5% (*P* < .05).

**ClinicalTrials registration number:** NCT04211870.

## 1 Introduction

Down syndrome (DS) is genetically characterized by the total or partial presence of an extra chromosome in par 21, also known as chromosome 21 trisomy. One of the manifestations found in individuals with DS is the muscular hypotonia, especially of the masticatory and oropharyngeal muscles. Such muscles may compromise basic actions such as swallowing, breathing and speech.^[1]^ Therefore, they may present alterations in oral functions, such as delayed eruption of deciduous dentition, open or crossbite, microdontia, macroglossia and reduced oral cavity, providing oral breathing and salivary leak.^[2]^

Hypotonia, muscle weakness, sluggish motor response information, and sensory changes can impair their ability to maintain adequate muscle balance. Consequently, the movements become difficult, slow and disorganized, resulting in stress, anxiety and consequently poor quality of life. In addition, due to muscle hypotonia, children with DS tend to project their tongue, and for more stable occlusion they also project their jaw. Associated, these factors may cause mouth breathing due to mouth opening, potentially causing sleep disturbance, Obstructive Sleep Apnea syndrome, and bruxism.^[3,4]^

Bruxism is defined by masticatory muscle activities that occur during sleep (characterized as rhythmic or non-rhythmic) and wakefulness (characterized by repetitive or sustained tooth contact and/or by bracing or thrusting of the mandible).^[5]^

Ethylogy is multifactorial and the main risk factors related to bruxism are gender, age, gene, mixed position, a lot of moving during sleep, sleeping with open mouth, snoring loudly, sleeping hours, sleeping anxiety, nervousness, passive smoking, high psychological reactions. with light on, cheek tone, emotional symptoms, mental health problems, birth weight, hyperactivity.^[6]^ In individuals with DS bruxism is often associated with sleep problems, snoring and obstructive sleep apnea.^[1]^

Stress has been reported to increase hypothalamus-pituitary-adrenal axis activity and alter the pattern of cortisol secretion, an important hormone used as a biomarker of psychological stress. Free cortisol determination can be easily measured in saliva. Stress sensitivity, as assessed by salivary cortisol, may be a psychological factor associated with bruxism.^[7]^

There are limited studies related to disease management in children. Treatment involves the control of anxiety and sleep, psychotherapy, physical therapy, and medications. Considering the high prevalence and difficulty in resolving this condition due to the lack of established treatment, any knowledge about bruxism is important and can assist in establishing better assessment and treatment strategies. The goal of treatment is to reduce physical, muscular and psychological tension. Therefore, a multidisciplinary approach involving physical therapy, pharmacotherapy, dentistry and psychology is required.^[8,9]^

Acupuncture has also been successfully used for the treatment of bruxism, achieving a reduction in the activity of the masseter and anterior temporal muscles as well as a reduction of anxiety.^[10]^ The stimulation of particular acupoints can promote muscle relaxation, thereby alleviating muscle spasms, inflammation and pain. Moreover, such stimulation leads to the release of hormones, such as cortisol and endorphins, thereby promoting an analgesic effect.^[11]^ Acupuncture point stimulation can be achieved with the use of needles, laser or electric current.^[12]^

Low Intensity Laser Therapy is a noninvasive and inexpensive treatment. Trigger point irradiation is an effective treatment for orofacial pain as well as reduction of swelling and hyperemia.^[13]^ However, there are no studies reporting the result of applying low intensity laser therapy on acupoints in children with DS and bruxism.

The aim of the proposed study is to analyze salivary levels of dopamine and cortisol and muscle activity before and after treatment with low-level laser therapy administered to acupoints in children with DS.

## 2 Methods

### 2.1 Study registration

The protocol for this study was registered ClinicalTrials.gov registration number: NCT04211870 on December 26, 2019. Available online: https://www.clinicaltrials.gov/ct2/show/NCT04211870.

### 2.2 Ethics committee

**Figure 1:**
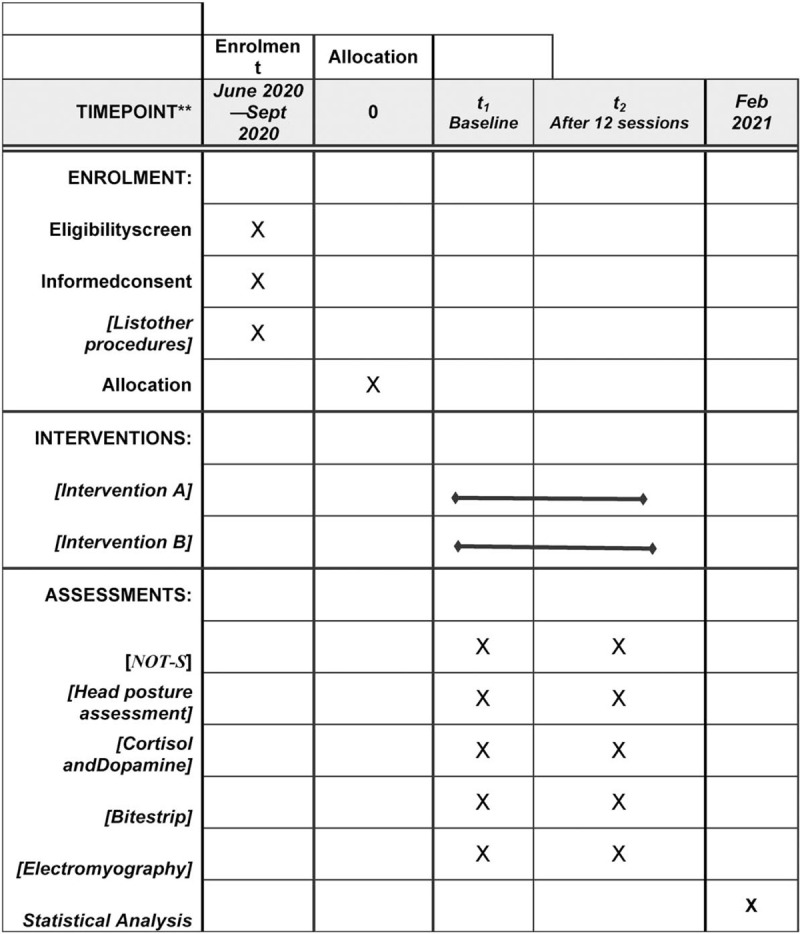
SPIRIT figure as recommended by 2013 SPIRIT Statement.

A randomized controlled trial will be developed following the schedule shown in Figure 1. The study will be conducted in accordance with the norms governing research involving human subjects stipulated in Resolution n° 466/12 and 510/2016 of the Brazilian National Board of Health and has received approval from the Human Research Ethics Committee of Nove de Julho University (certificate number: 3.726.654). The guardians will sign a statement of informed consent and the children and adolescents will also sign a consent form. The invitation to participate in the study will be made after a survey of patients on the waiting list of the Integrated Health Clinic of the university).

### 2.3 Participants

#### 2.3.1 Inclusion criteria

(1) Children and adolescents with a diagnosis of DS
(2) Four to seventeenyears of age
(3) Bruxism, based on parental reports of the occurrence of grinding the teeth, incisal and/or occlusal and the questionnaire for the evaluation of bruxism

#### 2.3.2 Exclusion criteria

(1) Use of muscle relaxant
(2) Temporomandibular disorder
(3) Undergoing other therapy for bruxism
(4) Other associated neurological diseases
(5) Cognitive deficit that impedes understanding the evaluations.

### 2.4 Evaluations

All evaluations will be performed before starting the treatment protocol and at the last session.

### 2.5 Nordic orofacial test - screening (NOT-S) protocol

The participants will be analyzed using the NOT-S protocol developed by a work group formed at the Second Nordic Conference of Orofacial Therapy in Gothenburg, Sweden in 2002. The NOT-S has been translated and culturally adapted to Portuguese and can be downloaded free of charge at www.mun-hcenter.se. Thisprotocol consists of a structured interview and clinical examination, each with six domains. The following functions are addressed during the interview:

(1) sensory function,
(2) breathing,
(3) habits,
(4) chewing and swallowing,
(5) drooling and
(6) dryness of the mouth.

The following functions are evaluated during the physical examination:

(1) face at rest,
(2) nose breathing,
(3) facial expression,
(4) masticatory muscle and jaw function,
(5) oral motor function and
(6) speech Each domain has 1 to 5 items reflecting the complexity of the specific function.

The NOT-S will be administered individually by the same examiner in an empty room with the child sitting erect. The interview portion will be performed using the questions on the screening form. For the evaluation of orofacial dysfunction during the clinical examination, the participants will be asked to perform tasks for each item in accordance with the illustrated manual. Each item has criteria for the respective function. A “YES” response or task the meets the criteria for compromised function will be scored 1 point, indicating dysfunction in the respective domain. A “NO” response or task that does not meet the criteria will be scored zero. The total is the sum of the points of all domains and ranges from 0 to 12, with higher scores denoting greater orofacial disfunction.

### 2.6 Head posture assessment

The clinical (visual) assessment of head posture will be performed using a posture grid. The participant will be instructed to stand with his/her natural posture. The natural balance of the head will be used to standardize the posture of each subject, who will be instructed to look forward with the gaze on the horizon. Semi-spherical polystyrene markers measuring 1.5 cm in diameter will be attached to the skin with double-sided adhesive tape at three anatomic points: the spiny process of the seventh cervical vertebra (C7), the manubrium of the sternum (A1) and the mental protuberance (MP). Photographs will be taken of each participant in right-side view.

### 2.7 Evaluation of salivary cortisol and dopamine levels

The participants and caregivers will receive verbal and written instructions to avoid any physical activity, the ingestion of substances with alcohol or caffeine, soft drinks, tea, corticoids, and chewing gum in the 24 hours before the collection of the saliva. Saliva samples will be collected using polyester swabs (Salivette, Sarstedt, Germany), which will be refrigerated immediately after collection. The swab will be placed under the tongue and the participant will be instructed to move it around the oral cavity until it becomes soaked with saliva (stimulated saliva. The swabs will be centrifuged at 3500rpm for 5 minutes. The supernatant will be collected and stored at −80°C.

Cortisol will be determined using an enzyme-linked immunosorbent assay (ELISA) (Salimetrics, State College, PA). Dopamine will also be determined using an ELISA kit (Dopamine Research ELISA BA E-5300). All kits were used following the manufacturer's instructions. The optical density of the samples was measured in a spectrophotometer at 450 nm.

### 2.8 Surface electromyography/diagnostic method (bitestrip)

*BiteStrip*is a disposable sensor that measures the activity of the masseter muscle during sleep.^[14]^

It can analyze contractions greater than 30% of maximum. Equipment parameters will be calibrated by C.C.B., durantedevide positioning and produces a classification categorized into four scores, depending on events number quantified in a five-hour period (A or L = less tha 30 events; B = 31 to 60 events; C = 61 to 100 events; D = more than 100 events). All vonlunteers will be instructed to use BiteStripdurang one sleepnigth at beginning and the end of study. Parents/guardian will be Orientated regarding left temporal muscle location and device uses of following manufacture's instructions.

### 2.9 Evaluation of electromyography of masticatory muscles

The electrical activity resulting from the activation of the muscles will measured using the six-channel TMJOINT electromyograph (BTS Engineering) with an bioelectrical signal amplifier, wireless data transmission and disposable bipolar surface electrodes (Ag/AgCl, Medical Trace) measuring 10 mm in diameter. The EMG signal will be amplified with a 2000-fold gain and filtered within a frequency of 20 to 450 Hz. The impedance and common rejection mode ratio of the equipment are >1015 Ω/0.2 pF and 60/10 Hz 92 dB. The data will be collected and digitized at 1000 frames/second using the BTS MYOLA software program.

After cleaning the skin with 70% alcohol to diminish the impedance, the surface electrodes will be attached to the belly of the muscle in the region of greatest tone (determined with the volunteer performance moderate intercuspation). The inter-electrode distance will be 20 mm center to center, as recommended by the European Society Recommendations for Surface Electromyography (SENIAM). A reference electrode will be placed on the left wrist of the volunteer to impede the interference of external noise.

The muscles analyzed will be the masseter (right and left) and anterior temporal (right and left). Evaluations will be performed under 4 conditions:

(1) at rest,
(2) during habitual maximum intercuspation with a strip of Parafilmbetween the molars to determine maximum voluntary contraction (MVC) of the muscles studied,
(3) during habitual chewing (isotonic contraction) and (IV) during maximum intercuspation (isometric contraction) without parafilm. All readings will be performed 3 times with a two-minute rest period between readings. The collection time will be 15 seconds with the muscle at rest, 5 seconds during MVC and 10 seconds during isotonic and isometric contraction. A metronome set at 60 beats per minute will be used for the reading during habitual chewing.

The EMG signal collected during chewing will be rectified and normalized by the mean of the signal followed by the calculation of the root mean square (RMS) using a 500-ms moving window without overlap. The data will be normalized by the highest RMS obtained during MVC.

### 2.10 Allocation

The participants will be randomized to two groups with the aid of a randomization site (randomization.com): Group A – active photobiomodulation(n = 15); Group B – sham photobiomodulation(n = 15).

### 2.11 Interventions

#### 2.11.1 Low-level laser therapy protocol

The same device (Laser DMC therapy EC) will be used for both groups. During sham treatment, however, the device will emit a guide light and sound, but no energy. Sessions will be held twice a week for six weeks (total: 12 session) using the parameters listed in Table 1. The participant will be seated comfortably in a quiet room with no sound interference. The tip of the laser will be covered in plastic wrap to avoid cross-contamination and for purposes of hygiene. The skin at the irradiation sites will be cleaned with gauze soaked in 70% alcohol.

**Table 1:**
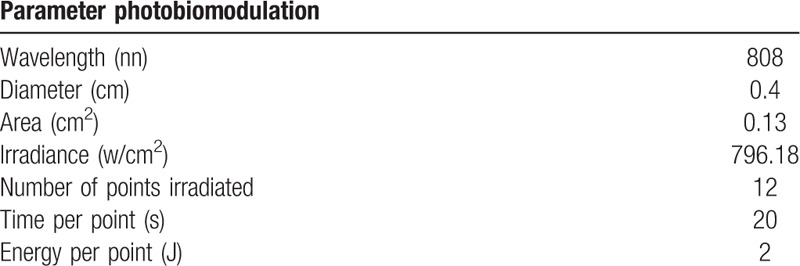
Parameters.

**Figure 2:**
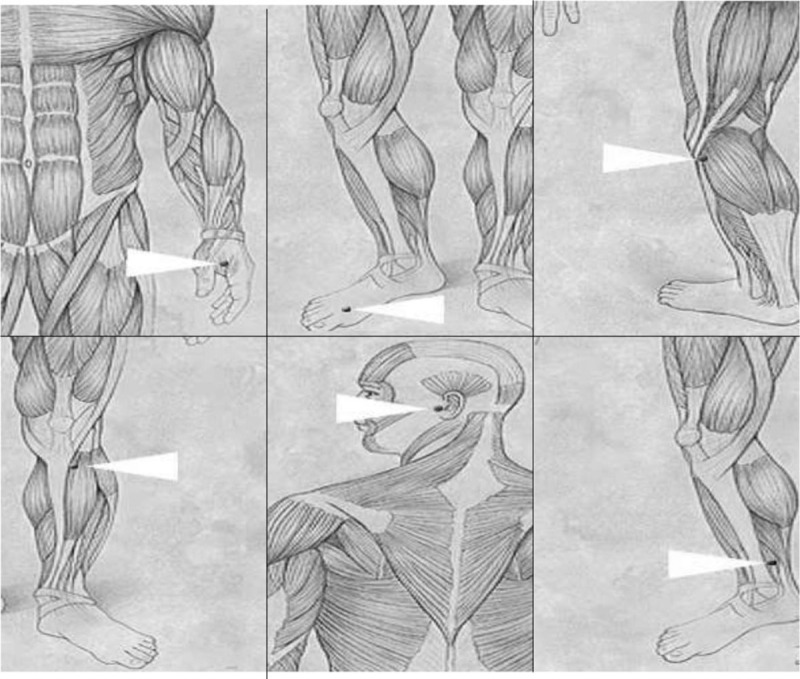
Acupuncture points will be irradiated - IG-4 (hegu) – On the radial side, between the 1st and 2nd metacarpal bone. This point exerts a strong, direct influence on the face, eyes, ears, nose and mouth. It is also used to calm the mind and alleviate anxiety.F-3 (taichong) – In the depression between the 1st and 2nd metatarsals near the metatarsal bases. Exerts a deep calming effect on the mind. Its calming action is enhanced when combined with IG-4. VB-34 (yanglingquan) – In the anterior and inferior depression of the head of the fibula. This is an important point to relax the tendons whenever there are muscle contractions. E-36(zusanli) – 3 cm below the patella and 0.5 cm to the side of the anterior edge of the tibia. Indicated for treating irritability, depression, insomnia, tiredness, fatigue and headache. ID-19 (tinggong) – In a depression anterior to the ear above the tragus. Indicated for treating problems in the ear region and temporomandibular joint dysfunction. BP-6 (sanyinjiao) – 3 cm above the prominence of the medial malleolus on the posteromedial border of the tibia. This is one of the most important points with a broad spectrum of action; exerts a strong calming effect on the mind and is generally used to treat insomnia.

A potentiometer will be used to measure the mean power of the equipment and therapeutic dose, ensuring safety to the participant and operator. Following the protocol suggested by Venezian et al^[13]^ and Carvalho et al.^[15]^(Fig. 2).

### 2.12 Statistical analysis

The data will be tabulated and treated using GraphPad PRISM version 7.0. The Kolmogorov-Smirnov test will be used to determine the normality of the data. Variables that fit the Gaussian curve will be expressed as mean and standard deviation. The ANOVA two-way will be used for comparisons between the groups, with the significance level set to 5% (*P* < .05). by one of the authors (PMC, Applied Statistics Specialist).

## 3 Discussion

The main objective of the study was to evaluate low intensity laser therapy administered to the use of acupuncture points in children with DS and bruxism. We hope that treatment with acupuncture points will be promising to improve bruxism symptoms in these children and cause a decrease in cortisol levels.

## Acknowledgments

To the University Nove de Julho (UNINOVE) for the availabilityof laboratories and volunteers.

## Author contributions

**Conceptualization:** Mônica da Consolação Canuto Salgueiro, Tamiris da Silva, Sandra Kalil Bussadori.

**Data curation:** Anna Carolina Ratto Tempestini Horliana, Olga Maria Altavista.

**Formal analysis:** Daniela de Fátima Teixeira da Silva, Alessandro Melo Deana.

**Investigation:** Tamiris da Silva, Elaine Marcilio Santos, Paula Midori Castelo, Carolina Carvalho Bortoletto.

**Methodology:** Tamiris da Silva, Mônica da Consolação Canuto Salgueiro, Andréa Oliver Gomes,

**Project administration:** Tamiris da Silva, Kristianne Porta Santos Fernandes, Sandra Kalil Bussadori.

**Resources:** Lara JansiskiMotta, Kritianne Porta Santos Fernandes.

**Software:** Marcela Leticia Leal Gonçalves, Anna Carolina RattoTempestini Horliana.

**Supervision:** Tamiris da Silva, Mônica da Consolação Canuto Salgueiro, Sandra Kalil Bussadori.

**Validation:** Tamiris da Silva, Daniela de Fátima Teixeira da Silva, Lara Jansiski Motta.

**Visualization:** Andréa Oliver Gomes, Marcela Leticia LealGonçalves, Daniela de Fátima Teixeira da Silva, Lara JansiskiMotta, Kritianne Porta Santos Fernandes.

**Writing – review and editing:** Andréa Oliver Gomes, Kristianne Porta Santos Fernandes, Raquel Agnelli Mesquita-Ferrari, Sandra Kalil Bussadori.

**Writing– original draft:** Mônica da Consolação Canuto Salgueiro, Tamiris da Silva, Raquel Agnelli Mesquita-Ferrari, Sandra Kalil Bussadori.
